# Significance of diet in chronic kidney disease

**Published:** 2013-07-01

**Authors:** Chaudhary Muhammad Junaid Nazar

**Affiliations:** ^1^Department of Endocrinology, University of Buckingham, Royal Gwent Hospital, NHS Trust, Wales, UK

**Keywords:** Diet, Chronic kidney disease, End stage renal disease, Nutrition, Diabetes mellitus

## Abstract

It is obvious that malnutrition is extremely dominant in end-stage renal disease (ESRD) patients. Malnutrition in pre-dialysis, dialysis, and post-dialysis stages is related to multiple factors. However, research work shows that if we try to improve the poor nutrition status of ESRD patients, good clinical outcomes may result. But the long-term effect of nutrition in the presence of other comorbid conditions has not been well established by many studies. So this aspect of nutrition is still researchable. Some studies emphasise that malnutrition is a major comorbid condition in ESRD victims as are hypertension, diabetes mellitus (DM) and cardiovascular disease. Researchers believe that the nutritional status, treatment and diagnostic parameters of these patients should be altered to achieve progress not only in their mortality outcome, but also in their quality of life

**Implication for health policy/practice/research/medical education:**

Researchers believe that the nutritional status, treatment and diagnostic parameters of chronic renal failure (CRF) patients should be altered to achieve progress not only in their mortality outcome, but also in their quality of life.

Diet plays an important role in patients with end-stage renal disease (ESRD) and a slight increase in any component of diet can make a major difference in pathogenesis of disease. Despite rapid progress in the science and technology of renal replacement therapy (RRT), the mortality rate of patients with ESRD remains high (1). Dietary interventions are essential in individuals with kidney diseases and nutritional recommendations vary depending on each patient’s stage of progression, cause of disease, medications and other treatment methods. During the past few years, the effect of diet in ESRD has been focused on in reasonably small studies. Protein-calorie malnutrition (PCM) is considered one of the most important risk factors among others that adversely affect outcomes in patients. During progression of chronic kidney disease (CKD), the requirements and utilisation of different nutrients change significantly. In addition, the presence of PCM is an important predictor for poor outcomes in these patients (2). Understanding the applicable nutritional principles and the available methods for improving the nutritional status of these patients is important. These changes ultimately place kidney disease patients at higher risk for PCM. In this review, I try to define the outcome of nutrition on ESRD patients. It focuses on the possible mechanisms that promote or cause poor nutritional status in ESRD patients. The treatment modalities and nutritional status are different depending upon on the risk factors relevant to each patient (3). These risk factors are discussed separately for chronic renal failure (CRF) patients yet to undergo RRT, dialysis or transplantation (including allograft). Also focussed on are measures to prevent malnutrition, as well as other treatment options in malnourished ESRD patients. While addressing this problem, what must be kept in mind is whether malnutrition is a simple reflection of multiple comorbidities such as hypertension, diabetes or CVD; or an independent risk factor that may aggravate existing comorbidities. There is no answer to this specific question of whether malnutrition is a separate comorbid condition or the outcome of existing comorbidity; hence it must be treated separately. However, recent studies show that the treatment of malnutrition improves the condition of patients and helps in reducing risk factors and fighting against comorbid conditions (4).

Chronic kidney disease (CKD) and associated risk factors

CKD is a disorder in which the kidneys lose their capacity to function. Normally, the functions of the kidneys are to filter the blood, produce urine, excrete wastes, and maintain electrolyte balance (5).

CKD is irreversible, progressive and a long-term condition. It may involve damage to both the kidneys. In the majority of patients, the main risk factors for CKD are hypertension and diabetes mellitus (DM) (4).

In the USA, the adjusted rate for new cases of ESRD reached 339 per million of the population in 2004, which was 21% more than in 1999 (3), while in the UK it affects one in every thousand of the populations (6). However, its occurrence is variable depending on dietary habits and different cultural values. CKD is more prevalent within South Asian and Afro-Caribbean communities as compared to the white population. The average age for developing CKD is 65, but it can be present at younger ages also (4). ESRD is the sternest form of CKD. It is characterized by severely reduced kidney function that is insufficient to maintain the kidney’s normal actions. Half of patients with ESRD are malnourished, which is associated with increased mortality rate. Life expectancy of ESRD patients can be improved by the use of RRT. The available options for RRT are dialysis or kidney transplantation. Dialysis was first started in the 1960s. There are two options for dialysis. Both procedures, hemodialysis and peritoneal dialysis, have benefits and some adverse effects, but definitely offer improved quality of life. The patients on dialysis may have symptoms such as shortness of breath, nausea, vomiting, poor appetite, weight loss, lethargy, confusion, itching, electrolyte imbalances, seizures and coma (Table 1). Of the two options, hemodialysis is mostly used, which involves the creation of a dialysis port, usually in the arm, and treatment at dialysis centers at an advised typical frequency of three times per week (6). The other option is continuous peritoneal dialysis, which involves inserting the dialysis material into the patient’s abdomen and allowing dialysis to occur continuously or intermittently without need for the patient to travel regularly to a dialysis center. Kidney transplantation is considered the more favorable method as compared to dialysis as it offers a greater number of beneficial and long-lasting effects. Early identification of a kidney replacement candidate is important so that necessary arrangement and planning can be done on time and that the patient can be referred to a nephrologist as early as possible. Renal transplantation offers a 5–10-year survival rate (6).

**Table 1:** Variables associated with decreased nutritional status of chronic kidney disease (CKD) patients

Increased protein and energy requirements
Losses of nutrients
Increased resting energy expenditure
Older age
Family history of CKD
Decreased protein and caloric intake
Anorexia
Frequent hospitalization
Inadequate dialysis dose
Comorbidities
Urinary tract disorders (e.g. kidney stones and urinary tract obstruction)
Systemic medical disorders: hypertension, diabetes mellitus, gastrointestinal diseases, ongoing inflammatory response–autoimmune disorders (e.g. systemic lupus erythematous)
Multiple medications
Non-steroidal anti-inflammatory drugs (e.g. ibuprofen) and contrast dye
Increased catabolism / decreased anabolism
Dialysis-induced catabolism
Amino acid losses
Induction of inflammatory cascade
Amino acid abnormalities
Metabolic acidosis
Hormonal derangements
Hyperparathyroidism
Insulin and growth hormone resistance

The primary complications that arise with most ESRD patients include hypertension, electrolyte imbalances (e.g. high potassium, low calcium, and high phosphate), excessive body fluids, and anemia. It is very important to manage complication early otherwise it may result in poorer clinical outcomes (7).

## Psychological issues in ESRD patients

Psychiatric disorders are common and can interfere with treatment. Depression is the most common psychiatric problem in ESRD patients. Devotion to suggested diet and fluid restrictions not only increases life expectancy, but also helps to reduce medical complications and treatment side effects and improve quality of life. Antidepressant treatment with medications and psychotherapy combined is not only effective in improving mood, but also improves nutritional status in dialysis patients (8). However, psychiatric disorders may interfere with treatment compliance.

Exercise training in ESRD patients is beneficial in many different aspects, especially against depression. It also reduces blood pressure and risk for cardiac arrhythmias and improves functioning of the heart (9). Therefore, exercise must be encouraged in CKD patients.

## Factors affecting nutrition in ESRD patients

Bearing in mind the greatness of the problem, one can predict that there are many risk factors playing significant parts in the development of malnutrition in ESRD patents. Many of these factors include comorbid conditions, hormonal factors, and lower intakes of protein, depression, and many more. Specific comorbid conditions can also facilitate the development of malnutrition in CRF patients. In the USA, DM is included among the leading causes of ESRD. But on the contrary, there is high incidence of malnutrition as compared to patients who are not diabetic, probably due to the multifactorial etiology. Diabetic patients are susceptible to malnutrition because of related gastrointestinal tract (GIT) symptoms such as gastroparesis and nausea insufficiency, as well as high manifestation of nephrotic syndrome and related complications (10).

There are some studies strongly suggesting that malnutrition is decreased intake of protein; these are observational and cross-sectional in design. The results from the feasibility phase as well as the full-scale MDRD study also provide important observations on this issue of filtration rate and caloric intake and reported protein intake. Thus, as an entry into study, it was found that the lower the GFR, the lower was protein and energy intake. The authors suggest that the signs of protein and caloric malnutrition become overt when GFR is less than 10 ml/min (13). A study was carried out to measure the effectiveness of dietary protein intake (DPI) in normal patients. The DPI of more than 90 patients was measured via urine collection at consecutive 24-hour intervals, measuring urine urea nitrogen excretion. Urine urea nitrogen excretion is considered as a rough indicator of DPI in normal patients. The results showed that numerous patients impulsively controlled their DPI at lower than 0.6 g/kg/day when creatinine clearance was less than 10 ml/min. Significantly, other markers of nutrition such as weight and IGF-1 concentration correlated with renal function as well as DPI (11).

Depression, which is commonly seen in ESRD patients, is also associated with anorexia. Socioeconomic status, lack of exercise, and old age are also other predisposing factors in the development of malnutrition in ESRD patients. Furthermore, CRF patients are usually recommended a large number of medications due to their multiple comorbid conditions; particularly sedatives, phosphate binders and iron supplements, which are also associated with gastrointestinal complications and mobility. All these mentioned factors also trigger the process of malnutrition (12). More recently, abnormality in the growth hormone/IGF-1 axis has been an important factor in the development of malnutrition in CRF patients. Growth hormone is the major promoter of growth in children and exerts several anabolic actions in adults, such as enhancements in protein synthesis, increases in fat mobilization and increases in gluconeogenesis, with IGF-1 as the major mediator of these actions (13). Although several studies have shown that plasma concentration of growth hormone (GH) actually increases during progression of renal failure, probably due to its reduced renal clearance, recent evidence suggests that uraemia per se is associated with the development of resistance to growth hormone action at cellular levels. Thus the current evidence suggests an interesting though not yet well-defined interrelationship between these hormonal, metabolic and nutritional factors involved in the evolution of malnutrition in ESRD patients (14).

Association of nutrition and outcome in kidney disease

A number of studies have evaluated the nutritional status of patients with advanced CKD (stages 3–5), reporting some degree of poor nutritional status. The prevalence of abnormalities has been estimated to range from approximately 20–60% of patients using various nutritional parameters. Similarly in stages 4–5, mild to severe malnutrition by subjective global assessment (SGA) is reported in 44% of patients, including 30% of patients on hemodialysis and 40% of patients on peritoneal dialysis. Serum albumin concentration has been identified as the most powerful indicator of mortality (12) and the low value of albumin and creatinine before dialysis has been considered an increased risk for mortality and morbidity during the past few years. A low value of serum albumin is considered to be 3.5–4.0 g/dl, which may increase the relative risk of death as compared to 4.0 g/dl or higher. On the other hand, decreases in creatinine (an indicator of muscle mass) and ideal weight have also been associated with increased risk of death in the patient population (10).

## Indices of nutritional status

Before discussing the importance of nutrition, it is important to discuss the different parameters being used by different medical practitioners to measure malnutrition. The parameters that have been proposed to assess nutritional status are albumin, cholesterol and creatinine, as well as more complex and not yet readily available parameters such as plasma and muscle amino acid profiles, pre-albumin, and insulin-like growth factor (IGF) (11). In addition, analysis of body composition will include different techniques, such as anthropometric and bioelectrical impedance measurements.

Serum albumin is the most extensively used method to measure malnutrition in CKD patients. It is an easily available marker but has a strong association with outcome. However, it is affected by the presence of other multiple co-existing factors in addition to malnutrition. Serum albumin decreases in response to any inflammation and thus may not reflect any change in nutritional status. It is also affected by the presence of other non-nutritional factors such as external losses (e.g. proteinuria), extracellular fluid volume and liver disease (14).

Low albumin is a reverse prognostic marker of survival in dialysis and post-transplant patients and is also often used as an indicator of malnutrition. Because its value as a stand-alone nutritional marker is unreliable, it should only be used in conjunction with other nutritional markers. The Renal Association recommends the regular measurement of serum albumin and suggests as a goal a level of over 30–35 g/l, depending on the lab assay used. Lower levels than this should prompt a clinical assessment of the patient for fluid overload, malnutrition, under-dialysis, or acute events such as infections (7).

The nutritional status of CKD patients can also be evaluated by daily protein intake (DPI). It is a very simple and direct method measuring dietary protein consumption. However, numerous studies indicate that this method lacks precision in estimating the actual intake of protein. However, there are also other suggested methods for calculating estimated protein intake, such as 24-hour urine urea nitrogen excretion in CKD patients or urea nitrogen appearance (UNA) rate calculation. However, these indirect assessments of DPI are only effective in stable patients and overestimate the actual intake in catabolic patients, in whom endogenous protein breakdown may lead to high UNA (15).

Anthropometric studies can be used for body composition analysis in CKD patients. More reliable and accurate methods of body composition analysis, such as prompt neutron activation analysis, which measures total body nitrogen content, and dual-energy x-ray absorptiometry, require expensive equipment and are available in only a few centers. A more recent proposed method is SGA, a simple method based on clinician experience to make overall assessment of nutritional status. The usefulness of SGA as a standard nutritional indicator in kidney disease has yet to be determined. Its advantage is that it is not expensive and includes objective data and several signs of poor nutritional status. Its disadvantages are full dependence on the clinical judgment of medical personnel and incompetence to tailor a specific nutritional intervention (Table 2) (7).

**Table 2:** Ideal-diet recommendations [modified from renal association guidelines (4)]

**Chronic kidney disease**	**Protein**	**Energy**	**Phosphorus**	**Sodium**
Stage 1–3 (GFR >30 mL/min)	No restriction	No restriction	600–800 mg/day	<2 g/day
Stage 3–5 (GFR <30 mL/min)	0.60–0.75 g/kg/day	35 kcal/kg/day	600–800 mg/day	<2 g/day
End-Stage Renal Disease	>1.2 g/kg/day	35 kcal/kg/day	600–800 mg/day	<2 g/day
Hemodialysis	>1.3 g/kg/day	35 kcal/kg/day	600–800 mg/day	<2 g/day
Peritoneal Dialysis	1.0–1.2 g/kg/day	35 kcal/kg/day	600–800 mg/day	<2 g/day
Acute renal failure	1.0–1.2 g/kg/day	35 kcal/kg/day	600–800 mg/day	<2 g/day

## Phosphate

High phosphate levels are a common problem among haemodialysis patients and contribute to cardiovascular disease and osteoporosis (16). High phosphate levels cause serum calcium to bind to it, leading to lower serum calcium levels as well as tissue and vascular calcium-phosphate calcifications (17). Low calcium levels lead to increased parathyroid hormone (PTH) production, which increases calcium absorption from the bone, resulting in osteoporosis. PTH production can be suppressed by 1, 25-dihydroxyvitamin D, the active form of vitamin D, metabolized in the liver and the kidneys. Patients with renal failure are not producing sufficient amounts of active vitamin D and PTH production is not sufficiently suppressed.

The renal association standards (RAS) recommends phosphate levels should be less than 1.8 mmol/l. Levels higher than this lead to high mortality rates. Much reduced levels are not possible to achieve without compromising suitable protein intake. Due to the high distribution volume and protein-binding capacity of phosphate, dialysis is of inadequate help in monitoring its levels. Even after dialysis, due to the characteristics mentioned, phosphate leads to rapid rebounds and makes dialysis useless. Dietary control alone can lead to malnutrition; phosphate is tied to protein intake (8). Most patients require the intake of phosphate binders to control phosphate levels while still achieving adequate nutrition.

## Potassium

Hyperkalemia and hypokalemia can have serious cardiac consequences for patients (7). Hypokalemia can lead to arrhythmias but is rare in chronic dialysis patients. Hyperkalemia is more common and causes reduced excitability of the cardiac cells and can in extreme cases cause sudden death due to asystole. The RAS suggests pre-dialysis potassium levels of 3.5–6.5 mmol/l. The use of low-potassium dialysate should be avoided. Levels should be dietary controlled and drugs causing an increase in serum potassium, such as angiotensin-converting enzyme (ACE) inhibitors, should be avoided (7).

In light of the above observation, it is strongly suggested that assessment of malnutrition must rely on dual parameters. Multiple markers used to assess malnutrition can reflect both somatic tissue from body composition analysis and visceral protein status from serum protein.

In summary, there are different methods available for assessing protein and energy nutritional status in CKD patients. Some are easy to perform, readily available, and inexpensive; whereas others are sophisticated, not available in many centres, and either expensive or have an unfavourable cost to benefit ratio. For example, a monthly nutritional screening can be easily performed in nearly any clinic or hospital by measuring serum albumin or pre-albumin, serum transferrin, and bioimpedance values. However, if the goal is to follow nutritional or body composition changes precisely and longitudinally, anthropometric and dual-energy x-ray absorptiometry methods may be useful (as well as even more sophisticated methods if available). For all indirect methods, repeated measures and technical standardisation are extremely important to reduce variability of results.

## Extent of malnutrition in ESRD patients

The extent of malnutrition in ESRD patients can be discussed at different levels, including pre-dialysis, dialysis and post-dialysis, and for transplant patients.

## Pre-dialysis patients

Malnutrition is not specific to any stage of ESRD, but evidently it is present even before starting RRT. Reports published by the MDRD study indicate that early signs of malnutrition—such as reduction in body mass index (BMI), weight and anthropometric measurements, and notable decline in urinary biochemistry parameters, including urinary creatinine excretion—were observed in CRF patients (10). Even similar reports were found favouring malnutrition before initiation of dialysis, indicating reductions in serum transferrin, serum cholesterol, serum IGF-1, percentage of body weight and urinary creatinine excretion as renal function deteriorated (15).

## Dialysis-dependent patients

### Hemodialysis

Once patients of CKD have been shifted from pharmacological treatment to RRT, the extent of malnutrition becomes more severe. Levy et al. reported serum albumin concentrations of less than 3.7 g/dl in 25% of their patient population, which included more than 12,000 haemodialysis patients (10). In the national cooperation dialysis study (NCDS), approximately 25% of patients on RRT were found to have insufficient dietary protein and energy intake, as well as up to 40% of the patient population exhibiting levels in body fat and muscle index lower than those predicted by total-body nitrogen (TBN) when analysed with PNAA analysis of body composition with different techniques has also shown evidence of malnutrition in chronic heart disease (CHD) patients (18).

### Peritoneal dialysis

Malnutrition is prevalent more in peritoneal dialysis patients than in haemodialysis patients. Some other studies also report high rates of malnutrition in continuous ambulatory peritoneal dialysis (CAPD) patients (19).

### Strategies for treatment of malnutrition in kidney failure patients

The energy requirement linked with chronic uremia is high, as is catabolic state. However, it is noted that patients who have been shifted from the pre-dialysis stage to RRT have been found to be still on a pre-dialysis diet. Patients on RRT are in need of a higher protein and calorie intake. Therefore, it is important to ensure that the dietary protein and calorie requirements of all patients are regularly checked. It is also clear that attempts should be made to encourage patients as well as their relatives to maintain adequate protein and calorie intake, especially after initiation of dialysis. Experienced dietitians must continuously check all ESRD patients’ diet in order to improve clinical outcomes. Also important is recognizing early signs of malnutrition. The visit of experienced dietitians should be made compulsory for outpatients’ settings; and equally for hospitalizations since these patients have even lower dietary protein and calorie intakes.

If patients are so ill or malnourished that they cannot eat anything, other options for energy supplementation must be considered. These are nasogastric feeding tubes and intra-dialysis parenteral nutrition (IDPN). Only a limited number of studies evaluating the effects of enteral supplementation in malnourished CKD patients have been done. Most of these are small in capacity and show only variable degrees of success (20). It is usually a tough task discerning whether an enteral supplementation is effective and when to try more expensive and invasive procedures such as IDPN.

Recent studies suggest that IDPN acutely improves net protein synthesis and increases albumin fractional synthetic rate. Several reports have highlighted the efficient use of IDPN as a conceivable therapeutic intervention in malnourished chronic dialysis patients. In a retrospective analysis of more than 1,500 chronic hemodialysis patients treated with IDPN, it was noted that long-term use of IDPN has been found useful and helped in decreasing mortality rate. On the other hand, studies using amino acid dialysate (AAD) in peritoneal dialysis patients have offered contradictory results. In studies proposing advantages from AAD, serum transferrin and total protein concentration increased and plasma amino acid profiles tended towards one or two exchanges of AAD per day. On the other hand, increases in blood urea nitrogen (BUN) concentration associated with exacerbation of uraemic symptoms as well as metabolic acidosis are potential complications of AAD (17). IDPN and AAD have been suggested as alternative methods of nutritional intervention in dialysis patients who cannot eat by the mouth. Unfortunately, studies evaluating the efficacy of nutritional procedures (IDPN and AAD) are subject to many design flaws. Therefore, their results cannot be trusted and more research work is still needed in order to evaluate efficacy, hence one should be very careful in prescribing expensive nutritional intervention.

### Transplant patients

The extent of malnutrition in transplant patients is still being researched and little is known about its effects. However, a few studies show that after transplantation, several parameters of patients were improved and that abnormalities in anthropometric measurements were observed in 38% of patients. One study indicates that transplant patients have some degree of depletion of visceral protein stores and decreased serum albumin concentration, specifically within the first year of transplantation. In one stable patient but with a transplant, researchers found a loss of muscle protein and also observed a reduction in muscle function (21). However, the actual prevalence of malnutrition, especially in patients with a kidney transplant, remains to be acknowledged and more research work must be encouraged to explore it further.

Several studies have encouraged patients with acute or chronic rejection to explore the importance of diet. However, the treatment and prevention of malnutrition has not been discussed about these patients. However, one can propose some intervention; especially in patients with acute rejection, the use of catabolic agents must be avoided in the early stages (22). However, in patients with chronic rejections, initiation of RRT is suggested for as early as possible, as are tapering dosages of corticosteroids.

## Author’s contribution

CMJN was the single author of the manuscript.

## Ethical considerations

Ethical issues (including plagiarism, misconduct, data fabrication, falsification, double publication or submission, redundancy) have been completely observed by the author.

## Conflicts of interests

There were no points of conflicts.

## Funding/Support

None.
